# A method to calibrate a camera using perpendicularity of 2D lines in the target observations

**DOI:** 10.1038/srep34951

**Published:** 2016-10-07

**Authors:** Guan Xu, Anqi Zheng, Xiaotao Li, Jian Su

**Affiliations:** 1 Traffic and Transportation College, Nanling Campus, Jilin University, Renmin Str. 5988#, Changchun, China; 2 Mechanical Science and Engineering College, Nanling Campus, Jilin University, Renmin Str. 5988#, Changchun, China

## Abstract

Camera calibration based on point features leads the main trends in vision-based measurement systems for both fundamental researches and potential applications. However, the calibration results tend to be affected by the precision of the feature point extraction in the camera images. As the point features are noise sensitive, line features are more appropriate to provide a stable calibration due to the noise immunity of line features. We propose a calibration method using the perpendicularity of the lines on a 2D target. The objective function of the camera internal parameters is theoretically constructed by the reverse projections of the image lines on a 2D target in the world coordinate system. We experimentally explore the performances of the perpendicularity method and compare them with the point feature methods at different distances. By the perpendicularity and the noise immunity of the lines, our work achieves a relatively higher calibration precision.

Camera is considered as an important instrument in the researches of three-dimensional reconstruction and computer vision^1^,^2^,^3^,^4^,^5^. The purpose of camera calibration is to obtain the transform parameters between the 2D image and the 3D space, which is crucial for the applications of biology^6^, materials^7^, image processing^8^,^9^,^10^,^11^, photon imaging^12^,^13^,^14^,^15^,^16^, physical measurement^17^,^18^,^19^,^20^, object detection^21^ and sensors^22^,^23^. A camera calibration system normally includes a CCD camera to capture the 2D image and a calibration target that is placed in the view filed of the camera in the 3D space. The measurement precision of the camera depends on the calibrated parameters. Therefore, it is significant to calibrate the camera with a precise approach.

The camera calibration is widely studied in recent years. It can be classified in three main categories, 3D cube-based calibration method, 2D plane-based calibration method and 1D bar-based calibration method. The 3D calibration methods are initially developed by Abdel-Aziz^24^. A 3D cubic target is established to provide the coordinates of the 3D points. The direct linear transform is invented to determine the transform matrix of the camera. Xu^25^ donated a three-DOF (degree of freedom) global calibration system to accurately move and rotate the 3D calibration board. A three-DOF global calibration model is constructed to calibrate the binocular systems at different positions. Then, the 2D plane-based methods^26^,^27^,^28^,^29^,^30^ are provided to promote the convenience of the on-site calibration and to simplify the target fabrication. A calibration method is proposed by Ying^31^ based on the geometric invariants. The camera parameters are solved by the projections of two lines and three spheres in the camera calibration. Shishir^32^ presented a method to calibrate a fish-eye lens camera. The camera is calibrated by defining a mapping between the points in the world coordinate system and the corresponding point locations in the image plane. Bell^33^ proposed a method to calibrate the camera by using a digital display to generate fringe patterns that encode feature points into the carrier phase. These feature points are accurately recovered, even if the fringe patterns are substantially blurred. Zhang^34^ outlined a camera calibration method that based on a 1D target with feature balls. The camera calibration is solved if one point is fixed. A solution is developed if six or more images of the 1D target are observed. Later, Miyagawa^35^ presented a simple camera calibration method from a single image using five points on two orthogonal 1D targets. The bundle adjustment technique is proposed to optimize the camera parameters. On the whole, although the accurate camera calibration is achieved by the 3D calibration targets, the precise 3D target is always difficult to be fabricated and carried. Moreover, it is difficult to apply in many fields due to the volume of the 3D target. The 2D methods are investigated to provide a convenient calibration compared with the 3D methods. A 2D calibration target contributes sufficient information of geometrical features to accurately calibrate the camera^36^,^37^,^38^. Besides, the 2D calibration target is in moderate size and easy to be fabricated. Although the 1D target takes the simplest model in geometry, the 1D calibration method generates less information compared with the 2D calibration method in one image.

A reliable method is outlined in this paper according to the 2D calibration target. As the point features are normally adopted in the 2D calibration method, the transform matrix is calculated by the relationship between the 2D projective lines in the image and the lines in the 3D space. Then, the reprojection errors of the lines are studied in order to verify the validity of the method. The point-based calibration methods are compared with the line-based method to evaluate the accuracy and the noise immunity in the calibration process. Finally, the lines on the target are reconstructed by the projective lines and the transform matrix with the camera parameters. The objective function is built and optimized by the perpendicularity of the reconstructed lines. The perpendicularity method is compared with the original methods to evaluate the performances of the approaches.

## Results

**Figure 1:**
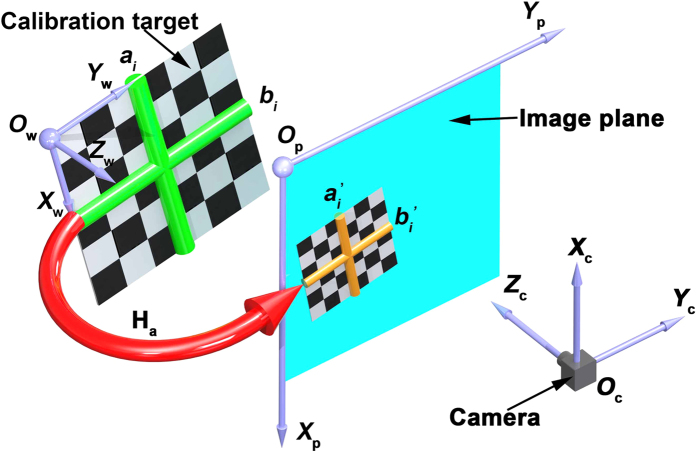
The method to calibrate a camera using perpendicularity of dual 2D lines in observations. *O*_w_-*X*_w_*Y*_w_*Z*_w_, *O*_p_-*X*_p_*Y*_p_ and *O*_c_-*X*_c_*Y*_c_*Z*_c_ indicate the world coordinate system, the image coordinate system and the camera coordinate system, respectively. H_a_ is the homography matrix from the world coordinate system to the image coordinate system. **a**_*i*_ and **b**_*i*_ are two perpendicular lines in the world coordinate system. 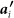 and 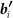 are the two projective lines of **a**_*i*_ and **b**_*i*_ in the image coordinate system.

In the process of the camera calibration, it is important to provide the relationship between the coordinates of the 2D image and the ones of the 3D space. A method is proposed for the camera calibration according to the perpendicularity of 2D lines in Fig. 1. A 2D target with the checkerboard pattern is adopted as the calibration object to construct the perpendicularity of 2D lines. The world coordinate system *O*_w_-*X*_w_*Y*_w_*Z*_w_ is attached on the 2D calibration board and the original point of the world coordinate system *O*_w_ is located at the upper left corner of the 2D calibration board. The axes *O*_w_*X*_w_ and *O*_w_*Y*_w_ are defined along two vertical sides of the target. The 2D calibration board is arbitrarily placed in the view filed of the camera. *O*_c_-*X*_c_*Y*_c_*Z*_c_ indicates the camera coordinate system. The original point of the camera coordinate system *O*_c_ is located at the optical center of the camera. *O*_c_*X*_c_, *O*_c_*Y*_c_ are respectively parallel to *O*_p_*X*_p_, *O*_p_*Y*_p_ of the image coordinate system. *O*_c_*Z*_c_ is the optical axis that is perpendicular to the image plane. **a**_*i*_ and **b**_*i*_ are two perpendicular lines generated from the checkerboard pattern on the 2D target. As the world coordinate system is attached to the 2D target, **a**_*i*_ and **b**_*i*_ are defined in the world coordinate system. 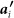 and 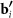 are the lines in the image coordinate system corresponding to the two perpendicular lines **a**_*i*_ and **b**_*i*_ in the world coordinate system. The transformation from the world coordinate system to the image coordinate system is a typical projective transformation; therefore, the line 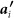 is not usually perpendicular to the line 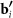 in the image. The vectors of the two lines 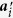 and 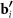 can be solved from the observations of the camera.

**Figure 2:**
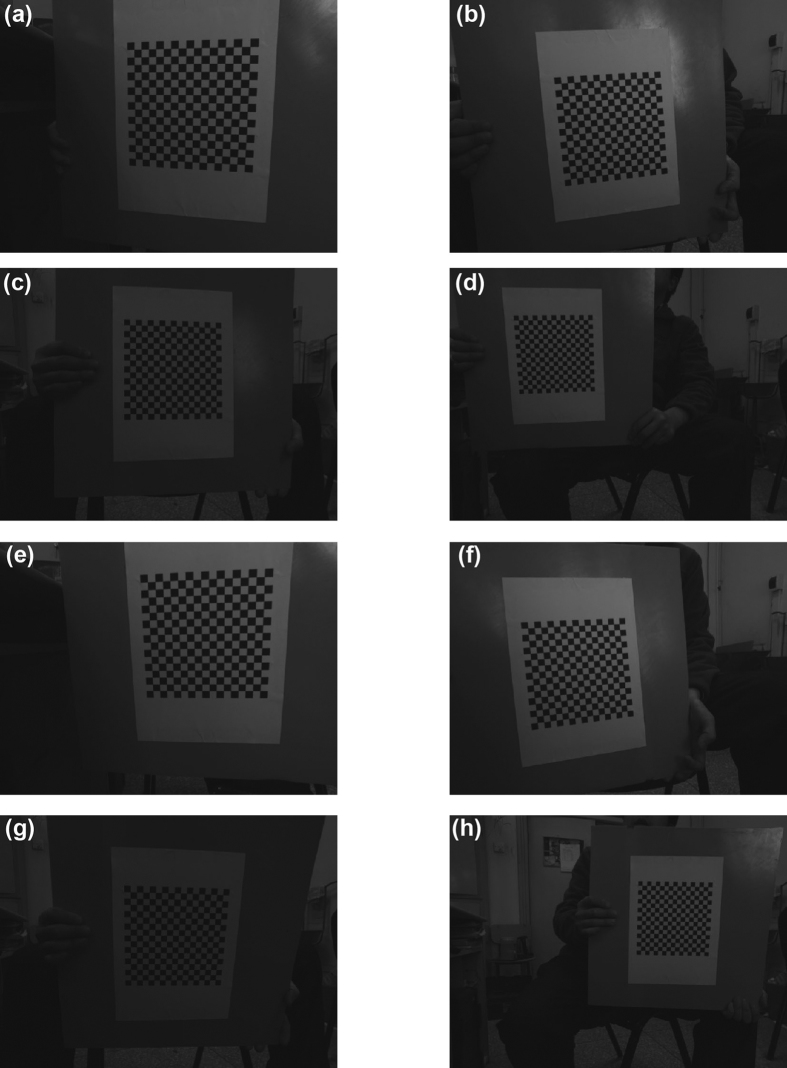
Two groups of the experimental images of the target with checkerboard pattern in the different distances. (**a**) The image at the distance of 400 mm in the first group experiments. (**b**) The image at the distance of 500 mm in the first group experiments. (**c**) The image at the distance of 600 mm in the first group experiments. (**d**) The image at the distance of 800 mm in the first group experiments. (**e**) The image at the distance of 400 mm in the second group experiments. (**f**) The image at the distance of 500 mm in the second group experiments. (**g**) The image at the distance of 600 mm in the second group experiments. (**h**) The image at the distance of 800 mm in the second group experiments.

According to the calibration method, the transform matrix H_a_ is generated from the 2D lines in the world coordinate system and the 2D projective lines in the image coordinate system, firstly. Then, the initial solutions of the intrinsic parameters of the camera are contributed by the transform matrices of the observations. Finally, the optimal solutions are provided by minimizing the objective function. The following experiments are divided to two aspects, the initial solution experiments and the optimal solution experiments, in order to verify the validity of the method based on the perpendicularity of 2D lines. The point-based methods proposed by Zhang^37^ and Tsai^39^ are adopted as the comparative methods. The two methods are 2D plane-based calibrations. An A4 paper with the checkerboard pattern is covered on the 2D target. The size of each square is 10 mm × 10 mm. Four capture distances, 400 mm, 500 mm, 600 mm and 800 mm, are chosen to study the effect of distance in the experiments, respectively. For each distance, ten images are captured to calibrate the camera. The resolution of the images is 1024 × 768. In the process of camera calibration, 32 lines are defined by the checkerboard paper in the world coordinate system. Figure 2(a–d) show the original images observed at the distances of 400 mm, 500 mm, 600 mm and 800 mm in the first group of images, respectively. Similarly, Fig. 2(e–h) are the second group of images at the distances of 400 mm, 500 mm, 600 mm and 800 mm, respectively.

**Figure 3:**
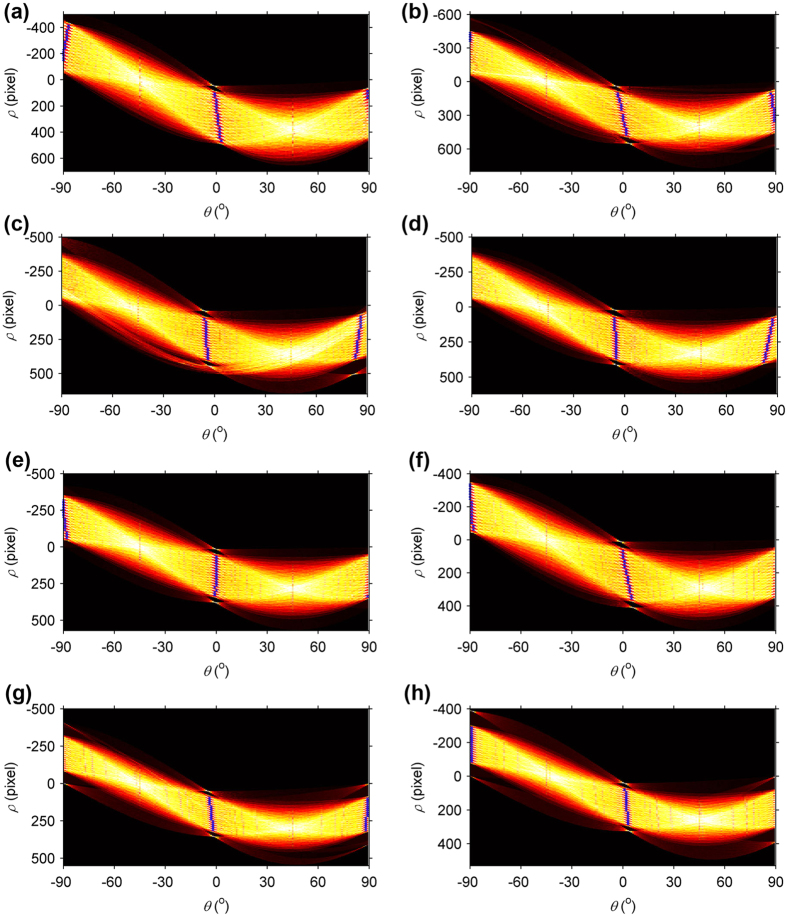
The results of the Hough transform in the polar coordinate system. The sinusoidal curves relate to the 2D points in the world coordinate system. The blue crosses represent the radial coordinates *ρ* and the angular coordinates *θ* of 32 lines in the polar coordinate system. (**a–h**) are the corresponding results to Fig. 2(a–h), respectively.

**Figure 4:**
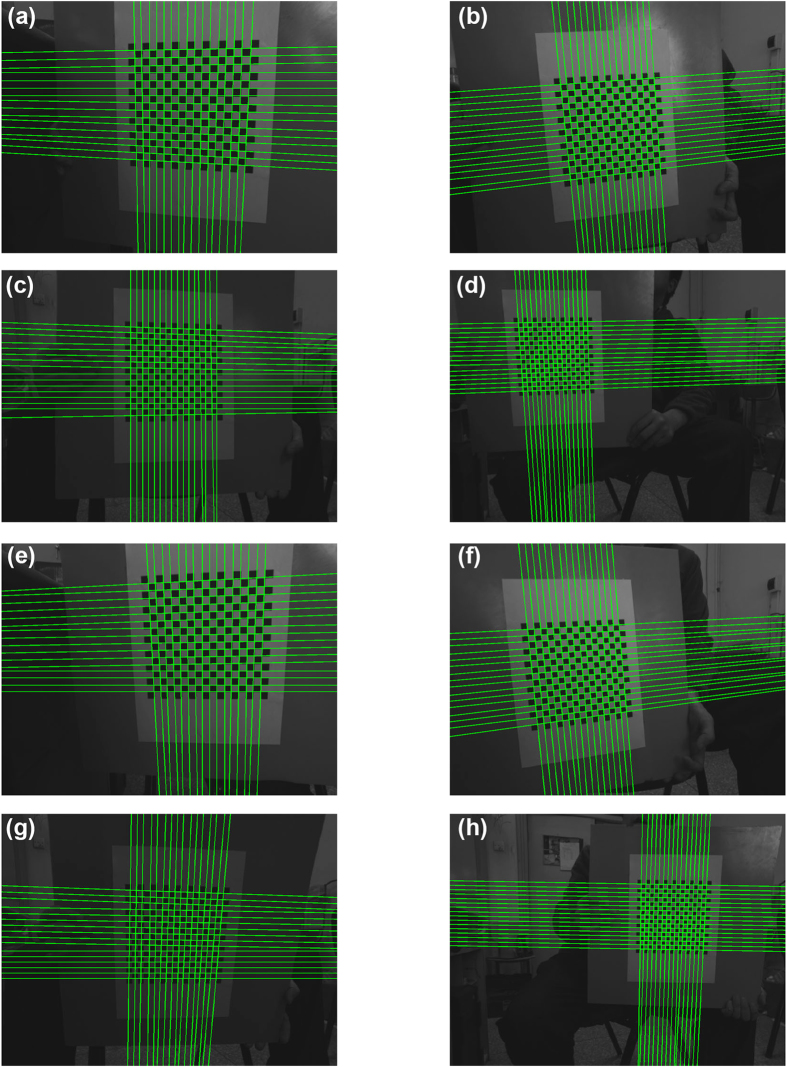
The recognition results of the 2D lines in the Cartesian coordinate system. The coordinates of the lines are derived from the radial coordinates *ρ* and the angular coordinates *θ* in the polar coordinate system. (**a–h**) are the corresponding results of Fig. 2(a–h), respectively.

The coordinates of the 2D projective lines are extracted by the Hough transform^40^,^41^,^42^ in the images. Hough transform finds the straight line in the parameter space that is less affected by noises. So the result of the line extraction is more stable than the result of the point extraction. Figure 3 shows the results of the Hough transform in the polar coordinate system. A sinusoidal curve corresponds to a 2D point in the Cartesian coordinate system. The blue crosses symbolize the radial coordinates *ρ* and the angular coordinates *θ* of 32 lines in the polar coordinate system. The extraction results of the lines are illustrated in Fig. 4(a–h). It is obviously that the Hough transform accurately detects the lines on the calibration target.

The line-based method is compared to the point-based methods to verify the calibration validity and noise immunity. The transform matrix H_a_ of the line projection is experimentally obtained from the coordinates of the lines in the image coordinate system and the coordinates of the lines in the world coordinate system. Then we have^43^


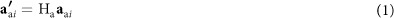


where ***a***_a*i*_ is the line in the world coordinate system, 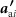 is the reprojection of the line ***a***_a*i*_ by the transform matrix H_a_. The transform matrices of the Zhang’s method and Tsai’s method are denoted by H_m_, H_t_. We have^43^


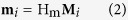



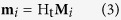


where **M**_*i*_ is the point in the world coordinate system, **m**_*i*_ is the point in the image coordinate system. The image points **m**_*i*_ are fitted to a straight line by the least square method. The coordinates of these lines are denoted by 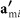, 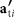. The errors of three methods are defined by


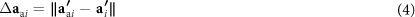



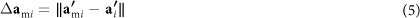



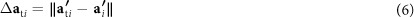


where Δ**a**_a*i*_ is the error of the line-based method, Δ**a**_m*i*_ is the error of the Zhang’s point-based method with 2D plane target, Δ**a**_t*i*_ is the error of the Tsai’s point-based method with 2D plane target, 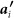 is the image line generated by the Hough transform, 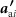 is the reprojection of the line **a**_a*i*_ by the transform matrix H_a_, 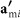 is the fitted line derived from the transform matrix H_m_ and the reprojections of points, 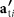 is the fitted line derived from the transform matrix H_t_ and the reprojections of points.

**Figure 5:**
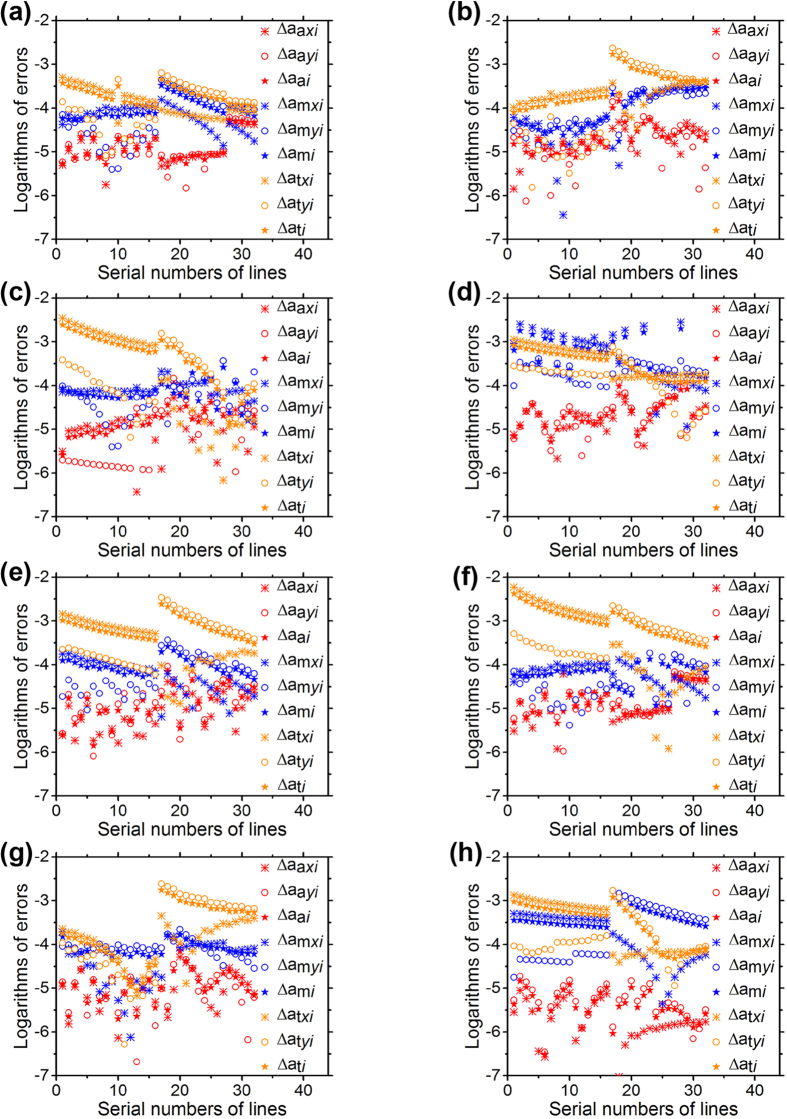
The errors of the reprojective lines adopting the line-based calibration method, Zhang’s and Tsai’s point-based methods in the *X* direction, *Y* direction and the root-mean-square of errors, respectively. (**a–h**) correspond to Fig. 2(a–h), respectively. The red data are the experiment errors of the line-based calibration method. The blue data are the Zhang’s point-based calibration method. The orange data are the Tsai’s point-based calibration method. The red data are located below the blue and orange data, which denote that the line-based calibration method contributes the higher accuracy.

The errors of the perpendicularity method, Zhang’s method and Tsai’s method are shown in Fig. 5(a–h). The errors of the line-based method are less than the point-based methods. The first group of experiments corresponds to Fig. 5(a–d). The mean errors and the variances are listed in Table 1. The second group of experiments corresponds to Fig. 5(e–h). The mean errors and the variances are listed in Table 2. According to the error data above, the errors in the *X* direction, *Y* direction and the root-mean-square errors of the line-based method are all far less than the errors of the point-based methods in the two groups of experiments. The errors of the line-based method vary indistinctively with the increasing distance. However, the errors of the point-based methods show the increasing trend when the distance is on the rise. The error variances of the line-based method have been compared with the error variances of the point-based methods. It is indicated that the variation range of the errors using the line-based method is smaller than the variation range of the point-based methods. The variances of the errors adopting the line-based method fluctuate in a small range with the increase of the distance. However, the variances of the errors using the point-based methods provide a significant jump with the increasing distance. The results reveal that the line-based method is less affected by the capture distance compared to the point-based methods. According to the results and analyses above, the line-based method contributes higher accuracy in the camera calibration process.

**Figure 6:**
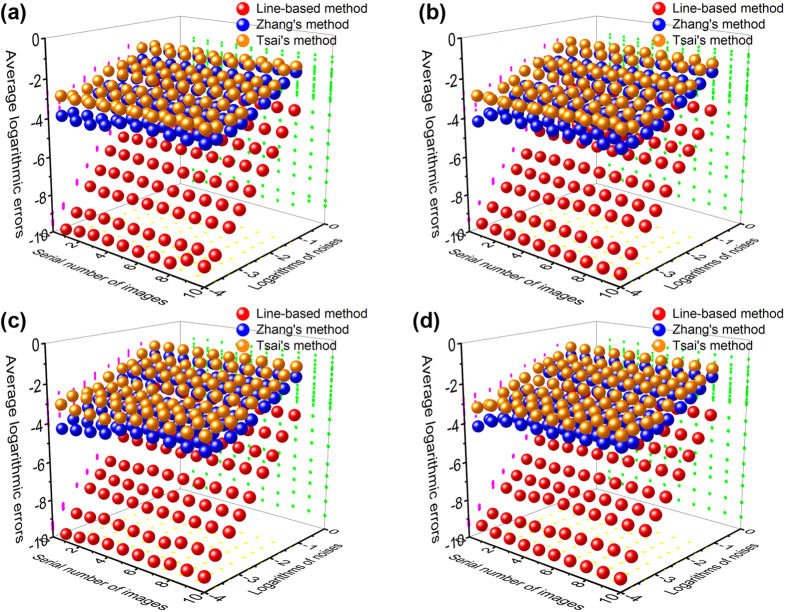
Average logarithmic errors of the line-based calibration, Zhang’s and Tsai’s point-based calibrations related to the logarithms of noises and the serial number of lines. (**a**) The comparison of the errors using the line-based method and the point-based methods at the distance of 400 mm. (**b**) The comparison of the errors using the line-based method and the point-based methods at the distance of 500 mm. (**c**) The comparison of the errors using the line-based methods and the point-based methods at the distance of 600 mm. (**d**) The comparison of the errors using the line-based method and the point-based methods at the distance of 800 mm.

The calibration accuracy of the three methods is further analyzed by adding different levels of Gaussian noises to the original images. The variances of the added noises are 0.0001, 0.0002, 0.0005, 0.001, 0.002, 0.005, 0.01, 0.02 and 0.05, respectively. The average errors are identified by the root-mean-square errors of the lines. The line-based method and Zhang’s and Tsai’s point-based methods are compared in Fig. 6. The values of the Gaussian noises are shown by the denary logarithms and the values of the average errors are presented by the natural logarithms for the purpose of direct observation. Figure 6(a–d) show the relationship between the average errors and the values of the noises at the distances of 400 mm, 500 mm, 600 mm and 800 mm, respectively. The errors of the three methods are all on the increase with the increasing noises. The average root-mean-square errors of the ten images using the line-based method increase from 9.06 × 10^−5^ to 4.10 × 10^−2^ as the noises vary from 0.0001 to 0.05 at the distance of 400 mm. Nevertheless, the average root-mean-square errors of the ten images based on the Zhang’s and Tsai’s methods are from 3.02 × 10^−2^ to 2.62 × 10^−1^ and from 5.14×10^−2^ to 3.15×10^−1^, as the noises vary from 0.0001 to 0.05 at the distance of 400 mm. Moreover, when the distance is 500 mm, the average root-mean-square errors of the ten images grow from 8.58 × 10^−5^ to 3.80 × 10^−2^. The average errors of Zhang’s and Tsai’s methods are from 2.81 × 10^−2^ to 2.52 × 10^−1^ and from 5.53×10^−2^ to 3.84×10^−1^, respectively. At the distance of 600 mm, the root-mean-square errors of the ten images using the line-based method increase from 9.88 × 10^−5^ to 4.08 × 10^−2^, respectively. The average errors of Zhang’s and Tsai’s methods are from 3.09 × 10^−2^ to 2.61 × 10^−1^ and from 5.54×10^−2^ to 4.20×10^−1^, respectively. At the distance of 800 mm, the root-mean-square errors of the ten images using the line-based method grow from 9.13 × 10^−5^ to 3.93 × 10^−2^, respectively. The root-mean-square errors of Zhang’s and Tsai’s methods are from 3.21 × 10^−2^ to 2.57 × 10^−1^ and from 6.07×10^−2^ to 3.85×10^−1^, respectively. It is evident that the average errors of the line-based method steadily increase as the noises are on the rise. However, the average errors of the line-based method grow more slowly than the errors of the point-based methods under the nine levels of noises. It proves that the line-based method provides a better noise immunity compared with the point-based calibration methods.

**Figure 7:**
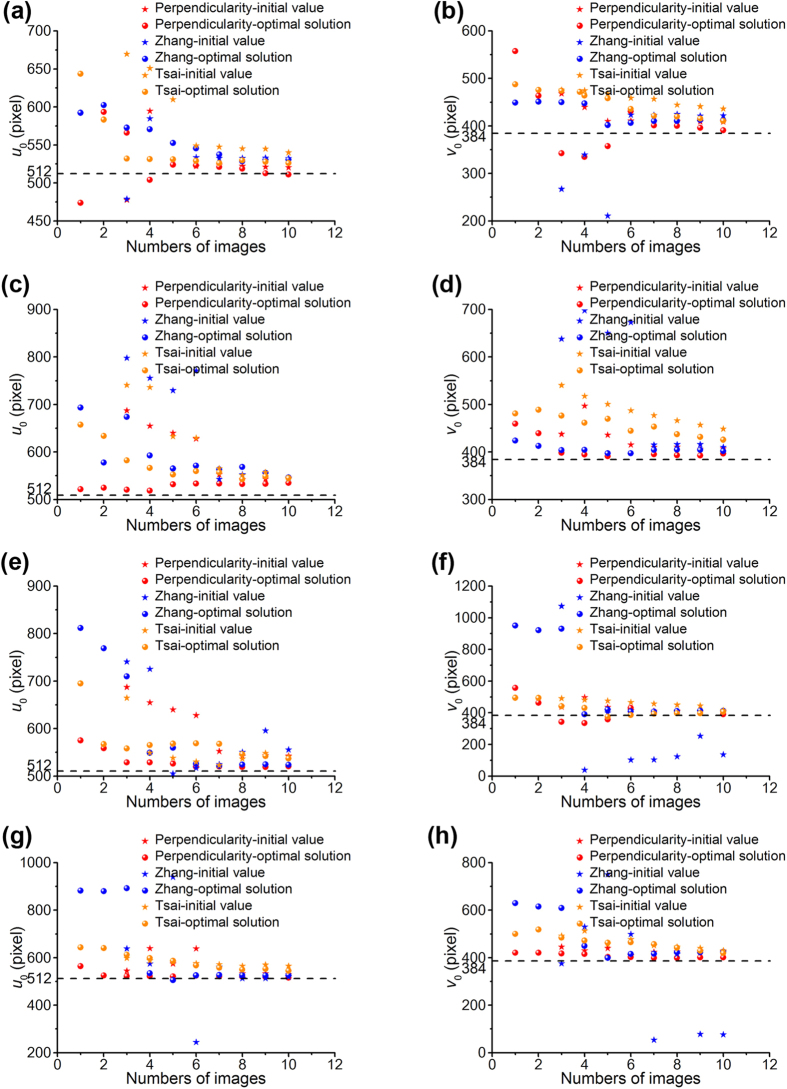
The calibration results of the principle point (*u*_0_, *v*_0_) generated from the perpendicularity method, Zhang’s method and Tsai’s method. The red marks show that the initial solutions and the optimal solutions of the perpendicularity method vary with the number of the images. The blue marks show that the initial solutions and the optimal solutions of Zhang’s method vary with the number of the images. The orange marks show that the initial solutions and the optimal solutions of Tsai’s method vary with the number of the images. (**a**) The initial and the optimal values of *u*_0_ of the perpendicularity method, Zhang’s and Tsai’s methods at the distance of 400 mm. (**b**) The initial and the optimal values of *v*_0_ of the perpendicularity method, Zhang’s and Tsai’s methods at the distance of 400 mm. (**c**) The initial and the optimal values of *u*_0_ of the perpendicularity method, Zhang’s and Tsai’s methods at the distance of 500 mm. (**d**) The initial and the optimal values of *v*_0_ of the perpendicularity method, Zhang’s and Tsai’s methods at the distance of 500 mm. (**e**) The initial and the optimal values of *u*_0_ of the perpendicularity method, Zhang’s and Tsai’s methods at the distance of 600 mm. (**f**) The initial and the optimal values of *v*_0_ of the perpendicularity method, Zhang’s and Tsai’s methods at the distance of 600 mm. (**g**) The initial and the optimal values of *u*_0_ of the perpendicularity method, Zhang’s and Tsai’s methods at the distance of 800 mm. (**h**) The initial and the optimal values of *v*_0_ of the perpendicularity method, Zhang’s and Tsai’s methods at the distance of 800 mm.

The calibration results are affected by noises in the camera calibration process. Therefore, the initial solution of the intrinsic parameters should be optimized to approach the real values of the parameters. The perpendicular method is proposed to solve the optimal value of the intrinsic parameter matrix. The elements of *u*_0_ and *v*_0_ indicate the principal point with pixel dimensions. As the principal point should theoretically coincide with the center of the image, *u*_0_ and *v*_0_ are chosen to evaluate the validity of the proposed optimal method. Figure 7 presents the optimal results of the initial values of the two elements *u*_0_ and *v*_0_ in the intrinsic parameter matrix. The dotted lines in Fig. 7 present the coordinates of the image center in the image coordinate system. Comparative experiments are performed on the perpendicularity method and Zhang’s method at the different distances.

According to Fig. 7, the initial values of *u*_0_ and *v*_0_ using the perpendicularity method approach to the coordinates of the center points of the images with the rising number of the images. The first few points are far away from the dotted lines. However, the optimal values of the perpendicularity optimal method are all near the dotted lines. The initial values of *u*_0_ and *v*_0_ based on Zhang’s and Tsai’s methods vary a lot with the increasing numbers of the images. Moreover, the optimal values are basically near the dotted lines as the number of the images is on the rise.

The means and the variances of the initial and optimal values of *u*_0_, *v*_0_ are listed in Table 3. The calibrated intrinsic parameters of the camera at the different distances are shown in Table 4. Considering the experiment data above, the mean values of the initial *u*_0_ and *v*_0_ adopting the perpendicularity method are more close to the coordinates of the center point compared with the Zhang’s and Tsai’s methods. The optimal values of *u*_0_ and *v*_0_ of the perpendicularity method show an obviously decreasing trend. The mean optimal values of *u*_0_ and *v*_0_ are close to the coordinates of the center point of the image. The optimal values of *u*_0_ and *v*_0_ based on the Zhang’s and Tsai’s optimal methods are also close to the coordinates of the center point. However, the descending velocity of the perpendicularity method is higher than the velocity of Zhang’s and Tsai’s optimal methods. Moreover, the variances of the optimal values of *u*_0_ and *v*_0_ based on Zhang’s and Tsai’s methods are larger than the variances of the perpendicularity method, except the variances of the initial and optimal values of *u*_0_ at the distance of 400 mm, the optimal value of *v*_0_ at the distance of 500 mm, the initial value of *u*_0_, the initial and the optimal values of *v*_0_ at the distance of 600 mm, and the initial value of *u*_0_ at the distance of 800 mm.

## Discussion

In the experiment results, the line-based calibration method contributes an initial solution with higher accuracy. The perpendicularity method describes a better optimization approach. In the camera calibration process, the coordinates of the geometrical features significantly affect the accuracy of the camera calibration. In the perpendicularity method, the Hough transform is employed to extract the coordinates of the lines. As the Hough transform takes the advantage of the higher noise immunity than the point extraction method, the coordinates of lines are more accurate than the coordinates of points for the geometrical features. Furthermore, the 2D lines are stable features with respect to the variable distance. However, the 2D points that are identified in a close observation are smaller in a far observation. As the noise effects are the same on the images, the point feature tends to be recognized in the different locations for different distances. Finally, as the lines pass though the feature points, the objective function adopts the perpendicularity of the lines as the optimal object, which includes more geometrical information than the feature point superposition.

## Methods

The previous method adopts points as the calibration features. Consequently, Harris corner detector^38^ is often chosen to extract point coordinates in the image to calibrate the camera. In this paper, the coordinates of the lines in the image coordinate system are generated from the Hough transform^40^,^41^. The coordinates of a random 2D point in the Cartesian coordinate system correspond to a sinusoidal curve with two parameters, the radial coordinate *ρ* and the angular coordinate *θ*, in the polar coordinate system. Thus, a line in the Cartesian coordinate system is transferred to a series of sinusoidal curves in the polar coordinate system by the Hough transform. The polar coordinates of the crossing point of the sinusoidal curves relate to the correct coordinates of the line in the Cartesian coordinate system. Thus, Hough transform extracts the line in the image by solving the optimal values of the radial coordinate *ρ* and the angular coordinate *θ* in the parameter space and transferring them to the Cartesian coordinate system.

The calibration method includes two procedures, initial solution and optimal solution. The initial solutions of the homography matrix H_a_ and camera parameters are solved at first by the similar way to the point-based calibration method^37^. The line transform from the 2D world coordinate system to the image coordinate system is represented as^43^


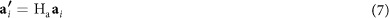


where **a**_*i*_ = [*a*_*i*_, *b*_*i*_, *c*_*i*_]^T^, 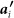 = [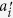, 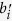, 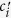]^T^, H_a_ = [**h**^1^ **h**^2^ **h**^3^]^T^, **h**^*j*T^ is the *j*^th^-row of H_a_, H_a_ is a 3 × 3 transfer matrix of the camera.

As the cross product of two same vectors, 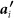 and H_a_**a**_*i*_, is a zero vector **0**, a 2D projective line 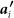 in the image coordinate system and 2D line **a**_*i*_ in the world coordinate system satisfy


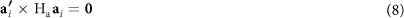


Equation (8) is rewritten as


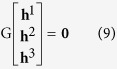


where 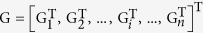, 
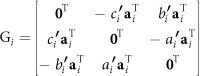
, 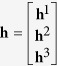.

The singular value decomposition of the matrix G is expressed by^44^


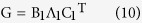


where B_1_ and C_1_ are orthogonal matrices, Λ_1_ is a diagonal matrix composed of the descending singular values.

From the right orthogonal matrix C_1_, we have^44^


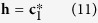


where 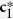 is the vector related to the smallest singular value in Λ_1_. H_a_ is obtained by arranging vector **h**.

The transform matrix H_a_ stands for the projection between 2D world lines and 2D image lines, however, the camera parameters have been calculated by the point-based transform matrix H_m_. Therefore, the transform from the line-based matrix H_a_ to the point-based matrix H_m_ should be performed on H_a_. Although the relationship between H_a_ and H_m_ are given by ref. 43, we investigate the relationship by the following another way.

Two 2D points **x**_1_ and **x**_2_ are projected to the image points 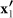 and 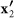 by the point-based transform matrix H_m_ as follows^43^


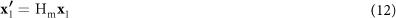



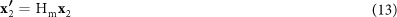


The 2D line **l** determined by the 2D points **x**_1_ and **x**_2_ is carried out as


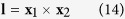


The projective line **l**′ determined by the image points 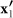 and 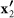 is


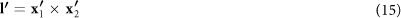


From equations (12), (13) and (15), the projective line **l**′ is


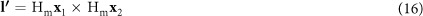


The right of equation (14) is transferred to


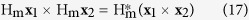


where 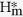 is the adjoint matrix of H_m_.

From equations (14), (16) and (17), we have


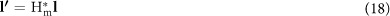


For a non-singular projective matrix H_m_, it is well known that


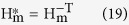


Stacking equations (7), (18) and (19), we obtain


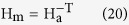


where 
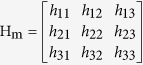
.

A projective matrix H_m_ is decomposed to^37^


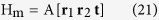


where H_m_ = [**h**_1_ **h**_2_ **h**_3_], **h**_*i*_ is the *i*^th^ column of H_m_, 
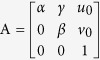
 is the intrinsic parameter matrix of the camera, (**r**_1_ **r**_2_ **t**) is the extrinsic parameters that relates the position and posture of the camera in the world coordinate system.

The intrinsic parameters of the camera can be solved by^37^


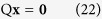


where 
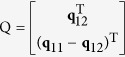
, **q**_*ij*_ = [*h*_*i*1_*h*_*j*1_, *h*_*i*1_*h*_*j*2_ + *h*_*i*2_*h*_*j*1_, *h*_*i*2_*h*_*j*2_, *h*_*i*3_*h*_*j*1_ + *h*_*i*1_*h*_*j*3_, *h*_*i*3_*h*_*j*2_ + *h*_*i*2_*h*_*j*3_, *h*_*i*3_*h*_*j*3_]^T^, **x** = [*x*_1_, *x*_2_, *x*_3_, *x*_4_, *x*_5_, *x*_6_].

The singular value decomposition of the matrix Q that is derived from several homography matrices H_m_ is expressed by^44^


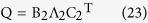


where B_2_ and C_2_ are orthogonal matrices, Λ_2_ is a diagonal matrix with the descending singular values. From the orthogonal matrix C_2_, we have^44^


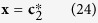


According to equation (24), the intrinsic parameters can be determined by^37^


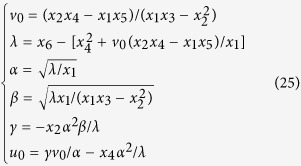


where *α*, *β* are the scale factors of the image, *γ* is the skew parameter of the image axes, *λ* is a scalar, (*u*_0_, *v*_0_) is the principal point with pixel dimensions.

The extrinsic parameters **r**_1_, **r**_2_ and **t** are obtained from equations (21) and (25) and given by^37^


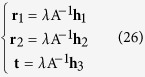


The intrinsic parameters in the matrix A are considered as the initial solutions of the camera. The image information is affected by noises, illuminations, capture distance and other factors. For this reason, an objective function is constructed to solve the optimal solutions of the camera parameters. The perpendicularity of lines is not preserved under the perspective imaging. However, the property is invariable to the reconstructed lines in the world coordinate system. The parameterized lines in the world coordinate system are reconstructed by the lines in the image coordinate system and camera parameters. Then, the objective function is established by the sum of the dot products among the perpendicular reconstructed lines. Finally, the camera parameters are achieved by minimizing the objective function.

According to equations (7) and (21), the relationship between the coordinates of the lines in the world coordinate system and the coordinates of the lines in the image coordinate system can be represented as^43^


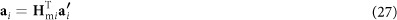



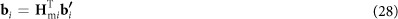


where 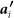, 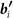 are the coordinates of the image lines in the *i*^th^ image, 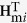 is the transpose of the point-based homography matrix of the *i*^th^ image, **a**_*i*_, **b**_*i*_ are the reconstructed lines in the world coordinate system.

The sum of the dot products among the perpendicular reconstructed lines should be theoretically zero, then


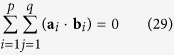


where **a**_*i*_ and **b**_*i*_ indicate a vertical line and a horizontal line in the world coordinate system, respectively.

From equation (21), H_m*i*_is written by the product of the intrinsic matrix and the extrinsic matrix as


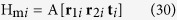


Stacking equations (27)-(30), 59, 63, 64, the objective function considering the perpendicularity of the reconstructed lines is given by


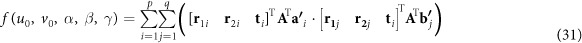


The optimal elements of the intrinsic parameters of the camera are obtained by minimizing the objective function and given by


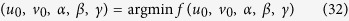


where arg means the arguments correspond to the minimized function *f*(*u*_0_, *v*_0_, *α*, *β*, *γ*,).

## Additional Information

**How to cite this article**: Xu, G. *et al*. A method to calibrate a camera using perpendicularity of 2D lines in the target observations. *Sci. Rep.* **6**, 34951; doi: 10.1038/srep34951 (2016).

## Figures and Tables

**Table 1:** The errors of the perpendicularity method, Zhang’s method and Tsai’s method correspond to Fig. 5(a–d).

Distance	Solution method		Errors in *X* direction	Errors in *Y* direction	Root mean square of errors
400 mm	Perpendicularity method	Mean	1.60 × 10^−5^	1.21 × 10^−5^	1.27 × 10^−5^
		Variance	1.32 × 10^−5^	1.79 × 10^−5^	1.55 × 10^−5^
	Zhang’s method	Mean	7.19 × 10^−5^	5.95 × 10^−5^	9.17 × 10^−5^
		Variance	3.58 × 10^−5^	1.14 × 10^−4^	5.07 × 10^−5^
	Tsai’s method	Mean	1.67 × 10^−4^	1.82 × 10^−4^	2.08 × 10^−4^
		Variance	1.29 × 10^−4^	1.67 × 10^−4^	9.48 × 10^−5^
500 mm	Perpendicularity method	Mean	7.24 × 10^−6^	1.39 × 10^−5^	1.22 × 10^−5^
		Variance	9.65 × 10^−6^	1.99 × 10^−5^	1.53 × 10^−5^
	Zhang’s method	Mean	5.07 × 10^−5^	6.05 × 10^−5^	9.04 × 10^−5^
		Variance	5.04 × 10^−5^	9.34 × 10^−5^	6.89 × 10^−5^
	Tsai’s method	Mean	2.23 × 10^−4^	4.77 × 10^−4^	4.21 × 10^−4^
		Variance	1.01 × 10^−4^	6.23 × 10^−4^	3.98 × 10^−4^
600 mm	Perpendicularity method	Mean	1.43 × 10^−6^	1.40 × 10^−5^	2.10 × 10^−5^
		Variance	1.39 × 10^−5^	4.14 × 10^−5^	2.83 × 10^−5^
	Zhang’s method	Mean	5.96 × 10^−5^	8.28 × 10^−5^	8.63 × 10^−5^
		Variance	1.22 × 10^−4^	9.50 × 10^−5^	1.04 × 10^−4^
	Tsai’s method	Mean	8.35 × 10^−4^	2.75 × 10^−4^	7.32 × 10^−4^
		Variance	9.86 × 10^−4^	3.68 × 10^−4^	6.32 × 10^−4^
800 mm	Perpendicularity method	Mean	1.26 × 10^−6^	1.40 × 10^−5^	1.40 × 10^−5^
		Variance	1.66 × 10^−5^	1.91 × 10^−5^	1.68 × 10^−5^
	Zhang’s method	Mean	6.22 × 10^−5^	3.45 × 10^−5^	6.30 × 10^−5^
		Variance	3.08 × 10^−4^	5.25 × 10^−4^	2.95 × 10^−4^
	Tsai’s method	Mean	4.54 × 10^−4^	1.97 × 10^−4^	3.73 × 10^−4^
		Variance	3.22 × 10^−4^	1.62 × 10^−4^	2.18 × 10^−4^

**Table 2:** The errors of the perpendicularity method, Zhang’s method and Tsai’s method correspond to Fig. 5(e–h).

Distance	Solution method		Errors in *X* direction	Errors in *Y* direction	Root mean square of errors
400 mm	Perpendicularity method	Mean	1.05 × 10^−5^	7.31 × 10^−6^	1.62 × 10^−5^
		Variance	1.22 × 10^−5^	3.17 × 10^−5^	2.10 × 10^−5^
	Zhang’s method	Mean	8.17 × 10^−5^	4.69 × 10^−5^	6.17 × 10^−5^
		Variance	3.65 × 10^−5^	7.29 × 10^−5^	5.16 × 10^−5^
	Tsai’s method	Mean	4.79 × 10^−4^	7.20 × 10^−4^	7.65 × 10^−4^
		Variance	4.20 × 10^−4^	8.73 × 10^−4^	5.01 × 10^−4^
500 mm	Perpendicularity method	Mean	9.10 × 10^−6^	8.72 × 10^−6^	1.07 × 10^−5^
		Variance	1.42 × 10^−5^	1.66 × 10^−5^	1.35 × 10^−5^
	Zhang’s method	Mean	3.71 × 10^−5^	8.18 × 10^−5^	7.85 × 10^−5^
		Variance	4.99 × 10^−5^	5.97 × 10^−5^	5.27 × 10^−5^
	Tsai’s method	Mean	1.29 × 10^−3.^	5.84 × 10^−4^	1.22 × 10^−3^
		Variance	1.56 × 10^−4^	5.40 × 10^−4^	9.37 × 10^−4^
600 mm	Perpendicularity method	Mean	1.98 × 10^−5^	1.83 × 10^−5^	2.25 × 10^−5^
		Variance	2.69 × 10^−5^	2.36 × 10^−5^	2.28 × 10^−5^
	Zhang’s method	Mean	4.07 × 10^−4^	2.20 × 10^−4^	4.77 × 10^−4^
		Variance	8.28 × 10^−4^	9.66 × 10^−5^	5.38 × 10^−4^
	Tsai’s method	Mean	1.68 × 10^−4^	6.07 × 10^−4^	4.65 × 10^−4^
		Variance	1.27 × 10^−4^	6.92 × 10^−4^	4.78 × 10^−4^
800 mm	Perpendicularity method	Mean	2.48 × 10^−6^	4.65 × 10^−6^	3.81 × 10^−5^
		Variance	2.47 × 10^−6^	4.82 × 10^−6^	3.66 × 10^−6^
	Zhang’s method	Mean	1.28 × 10^−4^	1.82 × 10^−4^	3.91 × 10^−4^
		Variance	9.91 × 10^−4^	4.79 × 10^−4^	2.45 × 10^−4^
	Tsai’s method	Mean	4.72 × 10^−4^	2.37 × 10^−4^	4.52 × 10^−4^
		Variance	4.42 × 10^−4^	3.69 × 10^−4^	3.14 × 10^−4^

**Table 3:** The means and the variances of the initial and optimal values of *u*_0_, *v*_0_ in the experiments.

Distance			*u*_0_		*v*_0_	
	Solution method		Initial value	Optimal value	Initial value	Optimal value
400 mm	Perpendicularity method	Mean	553.26	524.83	420.71	407.47
		Variance	83.60	32.96	65.54	21.76
	Zhang’s method	Mean	556.51	556.33	366.39	425.10
		Variance	73.54	26.85	85.10	21.06
	Tsai’s method	Mean	582.10	546.04	456.91	446.26
		Variance	53.38	38.32	15.25	28.83
500 mm	Perpendicularity method	Mean	599.10	528.58	427.51	459.37
		Variance	59.63	6.33	23.48	30.78
	Zhang’s method	Mean	590.88	656.13	539.52	405.62
		Variance	50.69	116.43	135.01	7.80
	Tsai’s method	Mean	619.50	574.75	486.91	457.13
		Variance	81.01	39.53	31.36	21.92
600 mm	Perpendicularity method	Mean	598.95	531.67	420.71	407.47
		Variance	59.78	19.41	30.75	65.54
	Zhang’s method	Mean	589.41	602.05	279.16	566.64
		Variance	92.99	114.77	340.10	253.39
	Tsai’s method	Mean	553.86	571.69	460.18	421.66
		Variance	45.59	44.89	24.19	43.01
800 mm	Perpendicularity method	Mean	569.35	525.75	428.50	408.09
		Variance	45.45	13.87	10.96	9.24
	Zhang’s method	Mean	562.97	632.77	347.25	480.63
		Variance	191.15	174.17	255.17	95.86
	Tsai’s method	Mean	576.96	585.59	463.31	465.06
		Variance	11.66	36.94	28.37	30.72

**Table 4:** Results of the perpendicularity method, Zhang’s method and Tsai’s method in the experiments.

Distance	Solution method		*u*_0_	*v*_0_	*α*	*β*	*γ*
400 mm	Perpendicularity method	Initial value	520.37	409.12	1070.42	1164.73	157.82
		Optimal solution	511.18	309.72	1117.33	1107.13	16.39
	Zhang’s method	Initial value	532.24	421.73	803.64	−805.16	−1.27
		Optimal solution	530.73	412.04	831.21	833.22	2.33
	Tsai’s method	Initial value	539.95	436.15	733.6	793.86	2.35
		Optimal solution	526.11	410.61	804.98	802.71	3.21
500 mm	Perpendicularity method	Initial value	544.84	406.84	1107.30	1116.11	111.14
		Optimal solution	535.02	397.06	1114.70	1104.21	16.47
	Zhang’s method	Initial value	545.48	410.03	797.01	−798.81	−1.08
		Optimal solution	546.41	402.09	829.13	831.23	3.35
	Tsai’s method	Initial value	543.01	448.54	544.28	542.74	6.59
		Optimal solution	545.32	425.94	552.60	550.19	3.96
600 mm	Perpendicularity method	Initial value	542.98	406.94	1136.12	1104.04	94.35
		Optimal solution	520.78	390.72	1126.67	1110.53	16.85
	Zhang’s method	Initial value	555.69	136.55	800.08	−801.67	−1.21
		Optimal solution	524.00	412.73	831.14	833.05	3.38
	Tsai’s method	Initial value	541.06	415.21	877.73	876.95	3.89
		Optimal solution	537.32	406.93	703.10	704.11	4.20
800 mm	Perpendicularity method	Initial value	530.57	426.79	1075.77	1110.98	103.68
		Optimal solution	516.43	401.25	1101.35	1096.41	17.27
	Zhang’s method	Initial value	519.57	76.79	799.98	−801.58	−1.09
		Optimal solution	526.65	425.35	831.84	833.73	3.18
	Tsai’s method	Initial value	565.74	430.32	935.26	934.37	10.36
		Optimal solution	545.32	419.67	880.26	879.48	9.89
